# Protein Interaction Analysis of Senataxin and the ALS4 L389S Mutant Yields Insights into Senataxin Post-Translational Modification and Uncovers Mutant-Specific Binding with a Brain Cytoplasmic RNA-Encoded Peptide

**DOI:** 10.1371/journal.pone.0078837

**Published:** 2013-11-11

**Authors:** Craig L. Bennett, Yingzhang Chen, Marissa Vignali, Russell S. Lo, Amanda G. Mason, Asli Unal, Nabiha P. Huq Saifee, Stanley Fields, Albert R. La Spada

**Affiliations:** 1 Comparative Genomics Centre, School of Pharmacy and Molecular Sciences, James Cook University, Townsville, Queensland, Australia; 2 Department of Pediatrics, University of California San Diego, La Jolla, California, United States of America; 3 Department of Pediatrics, University of Washington Medical Center, Seattle, Washington, United States of America; 4 Department of Genome Sciences, University of Washington Medical Center, Seattle, Washington, United States of America; 5 Department of Pharmacology, University of Washington Medical Center, Seattle, Washington, United States of America; 6 Department of Cellular & Molecular Medicine, University of California San Diego, La Jolla, California, United States of America; 7 Department of Neurosciences, University of California San Diego, La Jolla, California, United States of America; 8 Rady Children’s Hospital, La Jolla, California, United States of America; University of Dayton, United States of America

## Abstract

Senataxin is a large 303 kDa protein linked to neuron survival, as recessive mutations cause Ataxia with Oculomotor Apraxia type 2 (AOA2), and dominant mutations cause amyotrophic lateral sclerosis type 4 (ALS4). Senataxin contains an amino-terminal protein-interaction domain and a carboxy-terminal DNA/RNA helicase domain. In this study, we focused upon the common ALS4 mutation, L389S, by performing yeast two-hybrid screens of a human brain expression library with control senataxin or L389S senataxin as bait. Interacting clones identified from the two screens were collated, and redundant hits and false positives subtracted to yield a set of 13 protein interactors. Among these hits, we discovered a highly specific and reproducible interaction of L389S senataxin with a peptide encoded by the antisense sequence of a brain-specific non-coding RNA, known as BCYRN1. We further found that L389S senataxin interacts with other proteins containing regions of conserved homology with the BCYRN1 reverse complement-encoded peptide, suggesting that such aberrant protein interactions may contribute to L389S ALS4 disease pathogenesis. As the yeast two-hybrid screen also demonstrated senataxin self-association, we confirmed senataxin dimerization via its amino-terminal binding domain and determined that the L389S mutation does not abrogate senataxin self-association. Finally, based upon detection of interactions between senataxin and ubiquitin–SUMO pathway modification enzymes, we examined senataxin for the presence of ubiquitin and SUMO monomers, and observed this post-translational modification. Our senataxin protein interaction study reveals a number of features of senataxin biology that shed light on senataxin normal function and likely on senataxin molecular pathology in ALS4.

## Introduction

Senataxin is a large 303 kDa protein so named to signify its homology with the yeast protein Sen1p. Senataxin is a nuclear protein, and it contains a highly conserved DNA/RNA superfamily-1 helicase domain, indicative of its role in nucleic acid processing. Disease-associated mutations in the senataxin gene (*SETX*) fall into two distinct categories. Recessive mutations cause a severe form of ataxia, Spinocerebellar Ataxia Autosomal Recessive 1 (SCAR1– OMIM: 606002), which is also known as Ataxia with Oculomotor Apraxia 2 (AOA2); while dominant mutations cause a motor neuron disease known as amyotrophic lateral sclerosis type 4 (ALS4; OMIM: 608465) [1], [2]. As two distinct neurodegenerative conditions result from *SETX* mutations, senataxin is likely important for neuron survival [1], [3], [4].

ALS4 is an unusual familial form of ALS, with presumed disease penetrance of 100%. Age of disease onset varies widely, ranging from 5–63 years in the largest pedigree examined to date [2]. Pathologically, post-mortem examination has revealed atrophic spinal cords with marked loss of anterior horn cells and degeneration of corticospinal tracts, with an absence of sensory clinical signs or symptoms [2]. ALS4 patients were originally studied electrophysiologically, and noted to display hyperactive deep tendon reflexes and normal sensory function [2], [5]. Motor conduction studies revealed reduced evoked amplitudes and normal conduction velocity. Overall, the clinical picture indicates an essentially pure motor systems disorder typical of ALS.

Altered RNA processing is a known cause of neurodegeneration, as documented in spinal muscular atrophy (SMA) [6], and the fragile X syndrome of mental retardation [7]. The theme of altered RNA processing in motor neuron disease has been further emphasized by the recent discovery of roles for the RNA binding proteins TDP-43 (ALS10) [8], [9] and FUS (ALS6) [10], [11] in sporadic and familial ALS. Based on the strong conservation of the senataxin helicase domain, aberrant RNA processing is also likely to be a feature of ALS4 neurodegeneration. While the precise functions of senataxin are yet to emerge [12]–[18], senataxin mutations known to cause ALS4 are limited to three amino acid substitutions. In one very large ALS4 pedigree, we discovered that all 49 affected members carried a L389S substitution mutation [1]. Yet, while two other rare mutations were reported in other senataxin protein domains (R2136H and T3I) [1], the L389S mutation has emerged as the most common cause of ALS4 thus far [19], [20].

The L389S mutation resides in the middle of a domain with functional importance for both senataxin and yeast Sen1p [21], [22]. Interestingly, missense mutations, associated with both dominant ALS4 and recessive SCAR1/AOA2, are located in either the helicase domain or the amino-terminal protein-interaction domain [4]. In yeast, the amino-terminal domain is defined by amino acids 1–600, and is the minimal fragment required to interact with key proteins, including Rad2p, a deoxyribonuclease required in DNA repair; Rnt1p (RNase III), an endoribonuclease required for RNA maturation; but most prominently with Rpo21p (Rpb1p), a subunit of RNA polymerase II (RNAP II) [22]. Interaction of sen1p/senataxin with RNAP II is presumed essential to facilitate their critical role in transcription termination and to resolve transient RNAP II mediated R-loop structures via the conserved helicase domain [14], [15]. Given the high level of structural conservation between Sen1p and senataxin [3] [4], [23], and the fact that Sen1p can bind functionally important proteins via its amino-terminal domain, we used the first 650 residues of the senataxin protein to screen a human brain expression library by yeast two-hybrid (Y2H) analysis, and compared the results for wild type senataxin and L389S senataxin. Our results revealed key aspects of senataxin biology and yielded an unexpected interaction that may have relevance to ALS4 disease pathogenesis.

## Materials and Methods

### Human Brain cDNA Expression Library: Y2H Screen

We wanted to compare proteins found to interact with wild type senataxin, with those interacting selectively with the L389S mutant form of the protein. To achieve this, we screened a human brain expression library (Clontech) produced from cDNA sequences isolated from the brains of three Caucasian patients aged 41–61 years, and subsequently cloned into the activation domain of pGADT7-Rec vector by homologous recombination.

Using the Matchmaker GAL4-based system, two independent Y2H screens were undertaken: (i) screen 1 utilized wild type senataxin residues 1–650; and (ii) screen 2 utilized the same fragment but containing the L389S mutation. Both senataxin bait fragments were cloned into the binding domain vector, pGBKT7-BD. The wt and L389S mutant bait constructs were cloned from full-length cDNA expression constructs generated previously [3]. Y2H controls included: (i) determining that BD-*SETX*-wt and BD-*SETX*-L389S expression were not toxic to yeast; (ii) testing for non-specific auto-activation of reporter genes; (iii) non-interactor negative controls, pGBKT7-53 and pGADT7-Lamin; and (iv) positive control interactors, pGADT7-T and pGBKT7-53. These screens were performed using a mating strategy with the library pre-transformed into host strain Y187 [24], [25].

Selected clones thought to result from valid prey-clone interaction with senataxin (BD-*SETX*) were further tested in two ways: (i) the mating was retested with the putative interacting clones pre-transformed into the PJ69-4A strain; and (ii) the library clone was isolated and co-transformed with senataxin bait vector in a patch-selection/serial dilution strategy. The ‘patching’ strategy is based on first selecting the co-transformants on Leu^−/^Trp^-^ media. Then, single colonies were used to inoculate an overnight (O/N) culture, and from this we ‘patched’ four, 5-fold serial dilutions beginning with the stock O/N growth onto Leu^−/^Trp^−/^His^-^ selective media with 3 mM 3-AT. In addition to the wild type and L389S bait clones, we generated a third bait clone, the AOA2 mutant, W305C. We surmised this clone may represent a loss-of-function mutant form of senataxin protein.

### Blastn Analysis of Y2H Clone Containing BCYRN1 (NR_001568.1) Sequence

We took the DNA sequence from this ALS4 interacting Y2H clone and subjected it to alignment analysis. The 138 bp of DNA sequence is shown here: gat cta gag gcc gag gcg gcc gac atg ttt ttt ttt ttt ttt tcc ttt ttc tgg aga acg ggg tct cgc tat att gcc cag gca ggt ctc gaa ctc ctg ggc tca agc tat cct ccc gcc tct tag cct ccc tga gag.

This sequence was submitted to NCBI database search analysis: Name - nr; Description - Nucleotide Collection nt; Program - BLASTN 2.2.28. While the top alignment hit overall was Pan paniscus (Bonobo) ncRNA BC200 (AF067778.1), the top human hit was BCYRN1 (NR_001568.1). We then undertook a direct alignment using NCBI Blastn between the 138 bp clone sequence and human ncRNA BCYRN1. The portion of this clone encoding a peptide with homology to known primate proteins is translated here: ggg tct cgc tat att gcc cag gca ggt ctc gaa ctc ctg ggc tca agc tat cct ccc gcc tct → GSRYIAQAGLELLGSSYPPAS.

### SETX Expression Constructs

Expression constructs were based on PCR amplifying the amino-terminal *SETX* 1–650 aa’s (n-Senataxin) from our existing full-length *SETX* wt and L389S vectors [3]. We ruled out the possibility of using the original full-length expression constructs for these studies as they produce extremely low expression levels, even when using the Amaxa nucleofection transfection method [3]. In brief, PCR amplification utilized the following primers: forward primer, 5′-GGT ACC cca cca tgg att aca agg atg acg-3′ (Kpn1 site) and reverse primer 5′ GAA TTC cat tgg ttc ttt aga aaa tgt tgg gct g - 3′ (EcoR1 site). Flag epitope was already present in the template plasmid. We cloned first into the pCR®II-TOPO holding vector. Then by standard cloning, we directed the flag-tagged, n-Senataxin into the final expression vector, pcDNA3.1, and validated the coding sequences by DNA sequence analysis. For the GST-tagged senataxin expression construct we utilized the pGEX-4T-2 vector (GE, Life Sciences).

### Cell Culture, Transfection, Immunoprecipitation and Western Blot

HEK293 cells were obtained from the ATCC and cultured in DMEM media supplemented with 10% fetal bovine serum (FBS) at 37°C. Transfection of 293T cells was performed using Lipofectamine 2000 (Invitrogen) according to the manufacturer’s protocol in 6-well culture dishes.

24 hours post-transfection, cells were washed with room temperature (R/T) PBS and a second wash with PBS at 4°C. After gently removing the PBS wash, cells were lysed with 600 µl of RIPA buffer for 30 min at 4°C. A cell scraper was used to ensure efficient lysis. Lysates were syringed with a 26-gauge needle and spun at 10,000 rpm for 10 min at 4°C. 60 µl of each sample was collected as a control (Total Cell Lysate). In preparation for the anti-GFP immunoprecipitation (IP), we performed a pre-clear with protein A/G agarose magnetic beads to remove proteins binding non-specifically to beads. In brief, we washed 10 µl of 1∶1 ratio protein A/G beads with 1 ml RIPA, three times. Beads were resuspended in 10 µl RIPA per sample and then added to each sample, which was rotated for 30 min at 4°C. Supernatant was removed to a new tube by binding A/G beads to the magnetic column. Then to begin the anti-GFP binding, we added 5 µl suspension of anti-GFP antibody (magnetic beads) (ab69315). Samples were rotated O/N at 4°C. Unbound supernatant was removed by washing 3-times with 500 µl RIPA buffer at 4°C. Prior to gel electrophoresis protein separation, we added 100 µl of SDS loading buffer to beads and boiled for 10 min at 80°C. After removal of magnetic beads, IP-samples were further analysed by Western blot (WB).

Protein lysates were resolved using NuPAGE® Tris-Acetate gradient Gels (Invitrogen) and transferred to PVDF membrane (Sigma) by 1 hr of electroblot at 30 volts and blocked with 4% non-fat dry milk. To detect FLAG epitope, anti-FLAG M2 antibody (Sigma: F1804) was used (1∶5,000) in conjunction with anti-mouse HRP secondary antibodies (Sigma: A9044) (1∶10,000). The ECL Plus HRP detection kit (Amersham) was used for chemiluminescent detection. The use of 20 mM N-Ethylmaleimide (Sigma-Aldrich) was used to inhibit ubiquitinase and sumoylase enzyme activity.

## Results

### Senataxin Amino-terminal Protein Interactions Identified by Y2H Analysis

**Figure 1:**
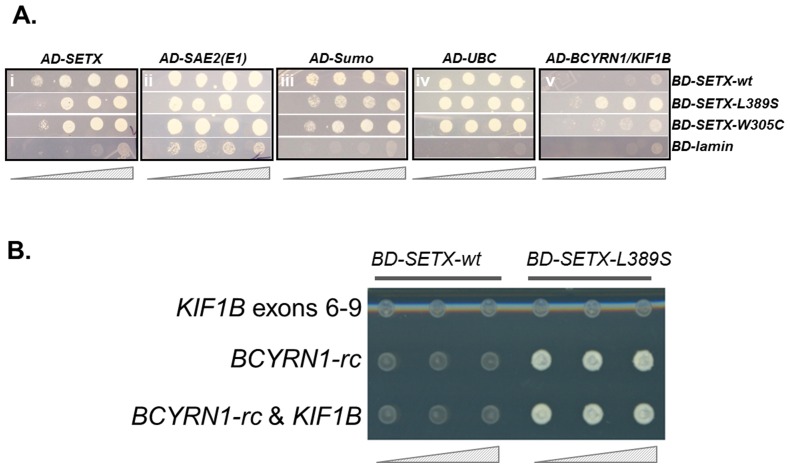
Validation tests of senataxin interactors identified from a human brain expression library. In (**A**) that follow, the four pGBKT7-BD bait vectors (BD-SETX-wt, BD-SETX-L389S, BD-SETX-W305C and BD-p53) were used in array with each pGADT7-AD test library prey clone: (i), senataxin; (ii), SAE2 (ubiquitin E1); (iii), Sumo; (iv), UBC and (v), KIF1B. Overnight cultures were plated 5-fold serial dilutions right to left (hatched triangle below). In (**B**), the library clone containing *KIF1B* and *BCYRN1*-rc cDNA sequences were sub-cloned to test which fragments were required for interaction as bait with: (i) the *KIF1B* coding exons 6–9; (ii) the 138bp of *BCYRN1*-rc; and (iii) the original full-length, ‘hybrid-clone’ was used as a positive control. The *BCYRN1*-rc sequence was sufficient to interact with N-terminal L389S senataxin. A 4-fold serial dilution (from right to left – hatched triangle) was undertaken for wt-*SETX* and L389S-*SETX* bait clones with each of the three prey fusion clones.

We prepared wild type senataxin as bait (DB-*SETX*-wt) and L389S senataxin as bait (DB-*SETX*-L389S), and proceeded to screen a human brain cDNA prey library. This Y2H analysis yielded 27 interacting clones for DB-*SETX*-wt and 57 interacting clones for DB-*SETX*-L389S in the initial two screens. Sequence analysis of the resultant clones revealed 18 unique transcripts, which were then re-tested (**Fig. 1**). Five false-positives were excluded from further analysis, as these clones also interacted with the negative control prey (pGADT7-Lamin), or interactions were not reproducible on re-testing. Most of the interacting library clones were full-length or nearly full-length, suggesting the library was of high quality. We categorized the 13 remaining validated clones into five groups (**Table 1**), as follows, based upon putative function.

**Table 1:** Y2H analysis for wt and L389S senataxin (1–650).

No	Group	Name	Function	NCBI ref	wt	L389S	W305C	Amino acids*
**1**	1	*SETX*	DNA/RNA helicase	NM_015046	+^1^	+^1^	+	1–668 N-term
**2**	2	*SAE2*	SUMO-1 activating enzyme subunit 2	NM_005499	+^2^	+^3^	+	451–641 ter
**3**	2	*Ubc9*	Ubiquitin-conjugating enzyme E2I	NM_003345	+^11^	+^31^	+	1–159 tot
**4**	2	*PIAS*	E3 SUMO-protein ligase	NM_016166	+^1^	+^3^	+	318–652 ter
**5**	2	*Sumo^†^*	Small ubiquitin-like modifier	NM_003352	+^2^	+^4^	+	1–102 tot
**6**	2	*UBC*	Ubiquitin C - gene encoding 9 repeats	NM_021009	+^2^	+^2^	+	1–532 tot
**7**	3	*EXOSC9*	Exosome component 9	NM_005033	+^2^	+^3^	+	158–440 ter
**8**	3	*TDG*	G/T mismatch- thymine DNA glycosylase	NM_003211	+^2^	+^3^	+	74–411 ter
**9**	3	*CHD3*	Chromodomain helicase DNA BP-3	NM_005852	+^1^	+^1^	+	1818–1967
**10**	3	*TOPORS*	Topoisomerase I binding	NM_005802	+^2^	+^3^	+	468–1046 ter
**11**	4	*HIPK2*	Homeodomain interacting protein-kinase2	NM_022740	+^1^	+^2^	+	216–527
**12**	5	*KIF1B*	Kinesin, anterograde transport	NM_015074	−^0^	+^1^	−	out of frame
**13**	5	*BCYRN1*	Homo sapiens brain cytoplasmic RNA 1	NR_001568	−^0^	+^1^	−	antisense

* Amino acids regions encoded by the representative clones are given for the wild type or mutant interacting clones that were used for subsequent validation retesting: ter = terminus; tot = total protein; and antisense indicates that the *BCYRN1* clone fragment was present in the 3′ to 5′ orientation. The number of independent clones obtained from the original wt and mutant library screens are listed as superscript numbers to the+in the ‘wt’ and ‘L389S’ columns, respectively. The SUMO clones (NM_003352) were isoform a, variant transcript 1 (***^†^***).

#### Senataxin self-interaction (Group 1)

We designated this group for senataxin self-interaction or dimerization. Both wild-type and L389S baits pulled out single, large independent clones from the initial library screens, which were subsequently validated by a range of assays (see below).

#### Ubiquitin/SUMO modifiers (Group 2)

We identified five interactors, each representing proteins in either the Ubiquitin protein degradation pathway or the related SUMO cascade, a moiety often used for protein trafficking [26]. Examples include SAE2, which is the E1-activating enzyme subunit 2 in the sumoylation pathway. SUMO is the small ubiquitin-like modifier added to the target protein and UBC is the human poly-ubiquitin protein. Interestingly, in addition to these Group 2 proteins with overt sumoylation functions, TDG (Group 3), TOPORS (Group 3), and HIPK2 (Group 4) have also been implicated in sumoylation pathway regulation – in addition to their defined primary functions.

#### DNA/RNA binding proteins (Group 3)

We observed a unique group of four DNA/RNA binding proteins involved in RNA surveillance, DNA repair, and other helicase functions. The EXOSC9 protein (exosome component 9) is part of the exosome, a multi-protein complex capable of degrading various types of RNAs. This 3′ to 5′ exo-ribonuclease complex is required for 3′ processing of 7S pre-rRNA to mature 5.8S rRNA that is localized to the nucleolus [27]. Thymine DNA glycosylase (TDG) fulfills the essential role of correcting G/T mismatches by a mismatch-specific DNA-binding glycosylase activity, thereby linking transcription and DNA repair [28]. TDG also exhibits sumoylation pathway function, as the SUMO binding activity of TDG is required prior to its covalent SUMO modification and subsequent colocalization with the promyelocytic leukemia protein (PML) within PML-containing nuclear bodies [29]. CHD3, the chromodomain helicase DNA-binding protein 3, is a central component of the nucleosome remodeling and histone deacetylase repressive complex (NuRD), and it has two chromatin organization domains (i.e. chromo-domains) and a helicase domain [30]. Topoisomerase I-Binding Arginine/Serine-Rich Protein (TOPORS) may perform a variety of complex functions, and TOPORS mutations cause autosomal dominant Retinitis Pigmentosa with perivascular retinal pigment epithelium atrophy [31]. TOPORS is also known as p53-binding protein 3 [32], and acts as a SUMO E3 ligase for p53 [33], in addition to serving as a binding partner for both SUMO-1 and SUMO-2 [34].

#### Kinase regulator (Group 4)

We identified a single protein known as the homeodomain interacting protein-kinase 2 (HIPK2). HIPK2 co-localizes and interacts with p53 and CREB-binding protein within PML nuclear bodies [35]. Interestingly, HIPK2 nuclear localization and function is mediated by a SUMO interaction motif [36], further underscoring the extent of sumoylation pathway interactome relationships existing among senataxin interactors identified in this Y2H screen. Activation of HIPK2 by UV-radiation leads to selective p53 phosphorylation, facilitating CBP-mediated acetylation of p53 and promotion of p53-dependent gene expression [35].

#### ALS4 specific interaction (Group 5)

We noted a unique and highly reproducible protein interaction specific to only L389S senataxin. The interacting clone contained KIF1B cDNA sequence, initially suggesting that it was *KIF1B*, a kinesin molecular motor protein previously mutated in a pedigree with neurological disease [37]. However, further DNA sequence analysis revealed that this clone consists of two cDNA fragments, likely fused during cDNA library construction. Specifically, this clone contains the major portion of Brain cytoplasmic RNA 1, *BCYRN1* (NR_001568), fused to a *KIF1B* cDNA fragment containing intron 5, exons 6–9, and ∼1.4 kb of intron 9. This interaction was not observed with the AOA2 mutant, DB-*SETX*-W305C, when used as bait.

### Senataxin Self-association is not Abrogated by the L389S Mutation

**Figure 2:**
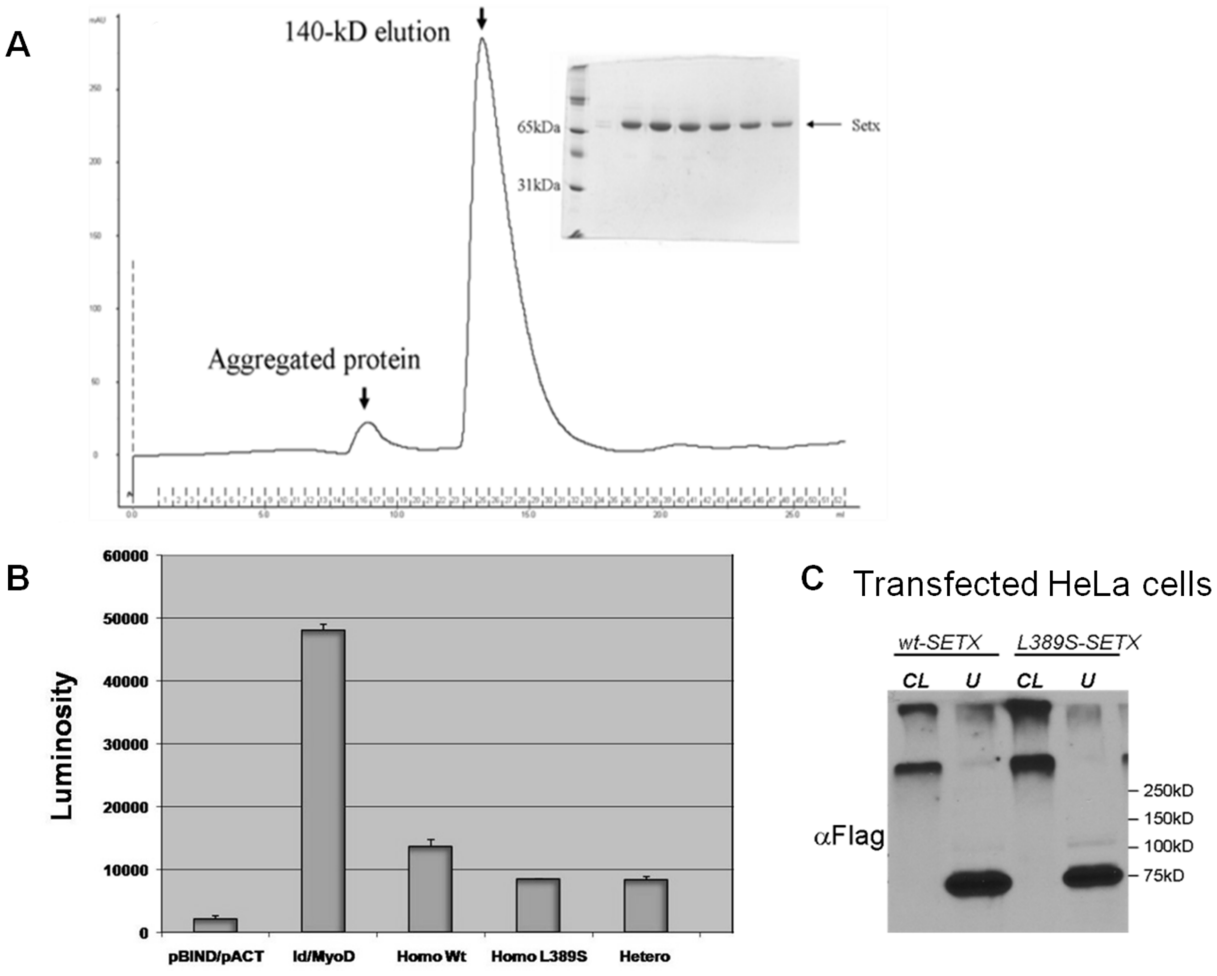
Biochemical assays showing that amino-terminal senataxin (1–650) seldom exists as a monomer, but is stable as a dimer. (**A**) Polyacrylamide gel electrophoresis was used to resolve purified senataxin peptide at ∼70 kDa, visualized by Coomasie stain. The filtration elution predicts a fragment size of ∼ 140-kD suggesting that the predominant form of purified senataxin exists as a dimmer. (**B**) Average luciferase activity (luminosity) for three transfection experiments. Column 1, pBIND/pACT (negative control); column 2, Id/MyoD (positive control); column 3, homo-wt [pBIND-SETX (1–650 wt) with pACT-SETX (1–650 wt)]; column 4, homo-L389S [pBIND-SETX (1–650 L389S) with pACT-SETX (1–650 L389S)]; and column 5, hetero [pBIND-SETX (1–650 wt) with pACT-SETX (1–650 L389S)]. (**C**) HeLa cells were transfected with Flag-tagged wt-SETX and L389S-SETX expression constructs. Compared with untreated HeLa cells (U), treatment with cross-linking (CL) reagent (DSS) caused 75 kDa bands to shift to greater than 300 kD with almost no monomer remaining.

ALS is one of a number of neurodegenerative proteinopathies in which protein aggregation may play a role in disease pathogenesis. As SOD1 aggregation in ALS1 may stem from impaired dimerization [38], we chose to examine the effect of senataxin mutation on its dimerization properties. To address this issue, we expressed GST-tagged senataxin (1–600) in *E. coli*, and after *in vitro* cleavage, we examined recombinantly produced amino-terminal senataxin by size-exclusion chromatography. Coomasie staining revealed the expected ∼70 kDa band for senataxin (1–600) (**Fig. 2A**, insert), while size-exclusion chromatography revealed a strong elution peak at ∼140 kDa (**Fig. 2A**), consistent with dimer formation. An initial smaller peak corresponding to a higher molecular mass was also noted, and may represent aggregated protein (**Fig. 2A**). To directly evaluate senataxin self-association, we cloned the amino-terminal senataxin cDNA (both –wt and –L389S) into the CheckMate mammalian two-hybrid (M2H) expression system (Promega). M2H analysis revealed strong interaction signals for *SETX*-wt homodimer, *SETX*-L389S homodimer, and *SETX*-wt – *SETX*-L389S heterodimer, though the self-association interaction appeared greatest for *SETX*-wt homodimer (**Fig. 2B**). To confirm these findings, we performed cross-linking studies using FLAG-tagged amino-terminal senataxin-wt and amino-terminal senataxin-L389S expression constructs in HeLa cells treated with disuccinimidyl suberate. Untreated HeLa cells expressing either *SETX*-wt or *SETX*-L389S displayed an intense band at ∼75 kDa on Western blot analysis, corresponding to senataxin monomer, as expected (**Fig. 2C**). When we immunoblotted transfected cells treated with the cross-linking agent, disuccinimidyl suberate, we observed two bands both migrating at >300 kDa for *SETX*-wt and *SETX*-L389S-expressing HeLa cells, without any senataxin monomer band present (**Fig. 2C**). The production of senataxin multimers by both *SETX*-wt and *SETX*-L389S confirmed that the L389S mutation does not prevent senataxin self-association.

### Senataxin is Subject to Ubiquitin – SUMO Post-translational Modification

**Figure 3:**
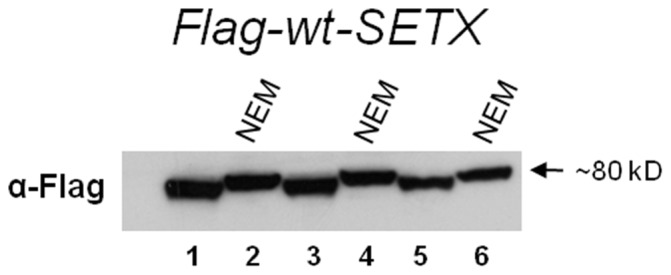
HEK293 cells are transfected with 1.5 µg (lanes 1, 2), 1.0 µg (lanes 2, 3) and 0.5 µg (lanes 5, 6) of Flag-wt-*SETX*. Note that in lanes, 2, 4, and 6 the transfected cells were treated with N-ethylmaleimide (NEM) to prevent the cleavage of either SUMO or ubiquitin and a band shift of ∼7 kDa is observed, suggesting that this is likely a sumoylation event.

The Y2H screen identified five interacting proteins that promote ubiquitination or SUMOylation (group 2), suggesting that senataxin is regulated by one or both of these pathways. Indeed, many redundant hits from both initial screens with wild-type senataxin and L389S senataxin were from this group. Previous studies with yeast Sen1p have documented that the amino-terminal region of Sen1p contains domains required for Sen1p degradation by the ubiquitin-proteasome system [39]. Although the basis of senataxin protein turnover is ill-defined, another possible role for ubiquitination and sumoylation is regulatory post-translational modification. To determine if senataxin is subject to such post-translational modification, we transfected FLAG-tagged senataxin-expression constructs into HEK293 cells, and then prepared cell extracts in the presence of NEM to inhibit ubiquitin and SUMO cleavage. We then performed Western blot analysis and noted a ∼7 kDa shift upward for all FLAG-*SETX*-wt protein isolated from NEM-treated HEK293 cell extracts (**Fig. 3**). A similar gel shift was observed with analysis of extracts from cells transfected with the FLAG-*SETX*-L389S construct suggesting the mutation does not affect this modification. Given the limitation of protein size estimates based on migration rates alone, this size increase is potentially consistent with the addition of a single ubiquitin or SUMO monomer.

### L389S-senataxin Binds to a Unique Peptide Encoded by BCYRN1-reverse Complement

**Figure 4:**
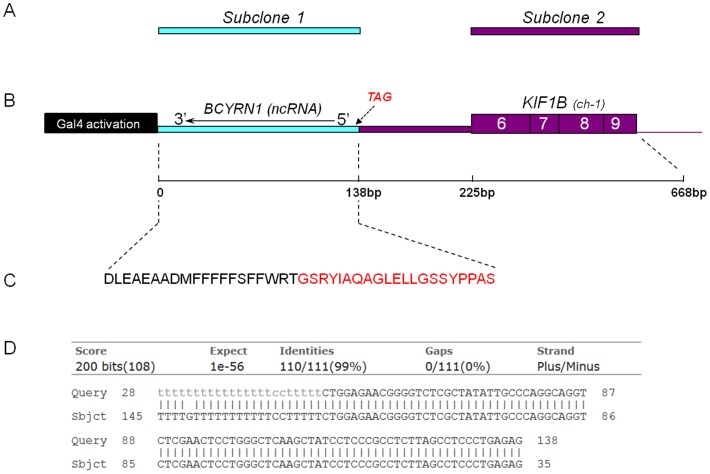
Structure of the brain cDNA library clone showing specific interaction with ALS4 L389S amino-terminal senataxin. Here we see a diagram of the fusion clone of *BCYRN1* reverse complement (*BCYRN1-rc*) and *KIF1B* cDNA sequences, with subclones of *BCYRN1*-rc and *KIF1B* coding exons 6–9 shown above the clone map (**A**), and relative length of relevant clone sequence in base pairs (**B**). Below is shown the minimal open reading frame peptide product capable of binding L389S senataxin, contained within the BCYRN1 sequence (**C**). The unique protein fragment highlighted in red text has near perfect homology with several human proteins with brain specific expression. When aligned by NCBI Blast, the 138 bp clone sequence (Query) shows 99% identity with BCYRN1 trancsript NR_001568.1 (Sbjct) over a 110 bp stretch (**D**).

Closer inspection of the fused *BCYRN1-KIF1B* clone revealed that the *BCYRN1* portion is oriented in the reverse complement direction (*BCYRN1*-rc) relative to the Gal4 activation domain, and DNA sequencing confirmed a stop codon immediately prior to the *KIF1B* cDNA sequence (**Fig. 4A**), ruling out expression of this gene product. We then separately subcloned *BCYRN1*-rc and *KIF1B* coding exons 6–9 into the pGADT7-Rec vector (**Fig. 4A**), and retested the L389S-senataxin bait construct for an interaction with these two subclones by Y2H analysis. We found that interaction with ALS4 L389S senataxin was dependent on *BCYRN1-rc*, but not on *KIF1B* coding sequence (**Fig. 4B**), a result that we robustly reproduced on repeated trials. A previous study identified *BCYRN1* as a non-coding RNA (ncRNA) gene that yields an ∼200 bp RNA, representing a primate neural-specific RNA polymerase III transcript [40]. BCYRN1 (NR_001568.1) spans ∼80 kb on chromosome Xq13.1, and is transcribed across 11 X-linked genes including the CMT1X (OMIM: 302800) peripheral neuropathy related gene, Connexin 32. Two related brain cytoplasmic RNAs, BC1 and *BCYRN1* (also known as BC200) have been shown to modulate gene expression at the level of protein translation [41]–[44]. This RNA class may represent a recently evolved transcript type predicted to regulate protein synthesis. *BCYRN1* derives from a transcribed monomeric *Alu* element which appears to have transposed from the signal recognition particle (SRP) RNA, and in particular from the SRP RNA region involved in translation arrest [45]. Although this regulatory RNA class is very novel and therefore remains uncharacterized, in normal aging, cortical *BCYRN1* levels typically decline and are reduced by >60% after age 48, but in Alzheimer’s disease brains, *BCYRN1* levels have been documented to increase [40].

**Figure 5:**
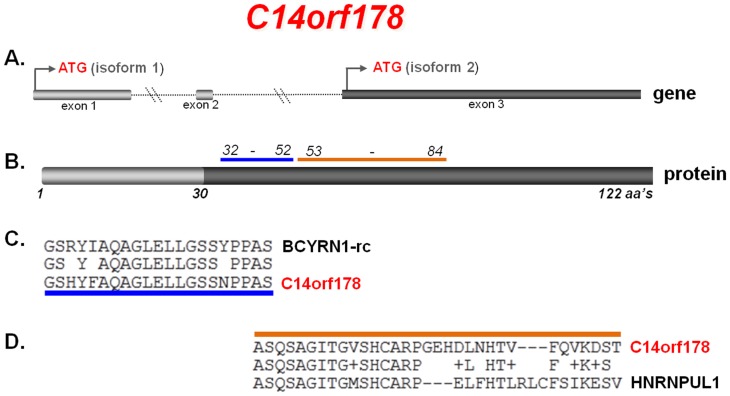
The 21 amino acid sequence which is translated from *BCYRN1-rc* (*fragment-21*) showed strong homology to two human proteins PPt1 and C14orf178. (**A**) The gene sequence of C14orf178, in which both isoforms contain *fragment-21*. (**B**) The larger protein encoded by C14orf178 isoform1. (**C**) The alignment of the *BCYRN1-rc* fragment with C14orf178. (**D**) The region of homology that C14orf178 isoform2 shares with the hNRNPUL-1 protein.

Following our NCBI Blastn analysis (**Fig. 4C–D**), our identification of a peptide product from the *BCYRN1-rc* sequence led us to consider the hypothesis that this translational product is genuine and not simply an artefact. One approach to evaluate the veracity of the *BCYRN1-rc* translational product is to scan the proteome for homologous proteins containing this domain. When we BLASTed the *BCYRN1-rc* peptide sequence against the NCBI non-redundant protein sequence database, we identified a 21 amino acid region GSRYIAQAGLELLGSSYPPAS, within the *BCYRN1-rc* coding sequence (**Fig. 4C**), that yielded highly significant hits for a large number of proteins, all restricted to the proteomes of primates. For humans, we found two proteins with significant homology: Palmitoyl-protein thioesterase 1 (PPT1) (NM_000310) and C14orf178 (NM_174943). The level of identity between the *BCYRN1-rc* peptide and the PPT1 protein was 81%, while the level of identity with the C14orf178 protein was slightly higher at 86%. PPT1 protein is a small glycoprotein involved in the catabolism of lipid-modified proteins during lysosomal degradation, and mutations in the PPT1 gene cause infantile neuronal ceroid lipofuscinosis 1 (CLN1; OMIM #256730) and neuronal ceroid lipofuscinosis 4 (CLN4; OMIM #204300). The C14orf178 gene encodes two small proteins of 92 amino acids and 122 amino acids, based upon its two predicted RNA isoforms (**Fig. 5A**). Both predicted proteins are homologous to the *BCYRN1-rc* peptide (**Fig. 5B–C**). The C14orf178 gene consists of 3 exons, spanning ∼9 kb of genomic sequence (NCBI UniGene Hs. 375834). Although the C14orf178 gene encodes two protein products, their functions remain unknown. Interestingly, the C14orf178 protein contains a 32 amino acid domain with 61% homology to the heterogeneous nuclear ribonucleoprotein U-like 1 protein (HNRNPUL1) (**Fig. 5D**).

### L389S-senataxin Interacts with the C14orf178 Protein Product in HEK293 Cells

**Figure 6:**
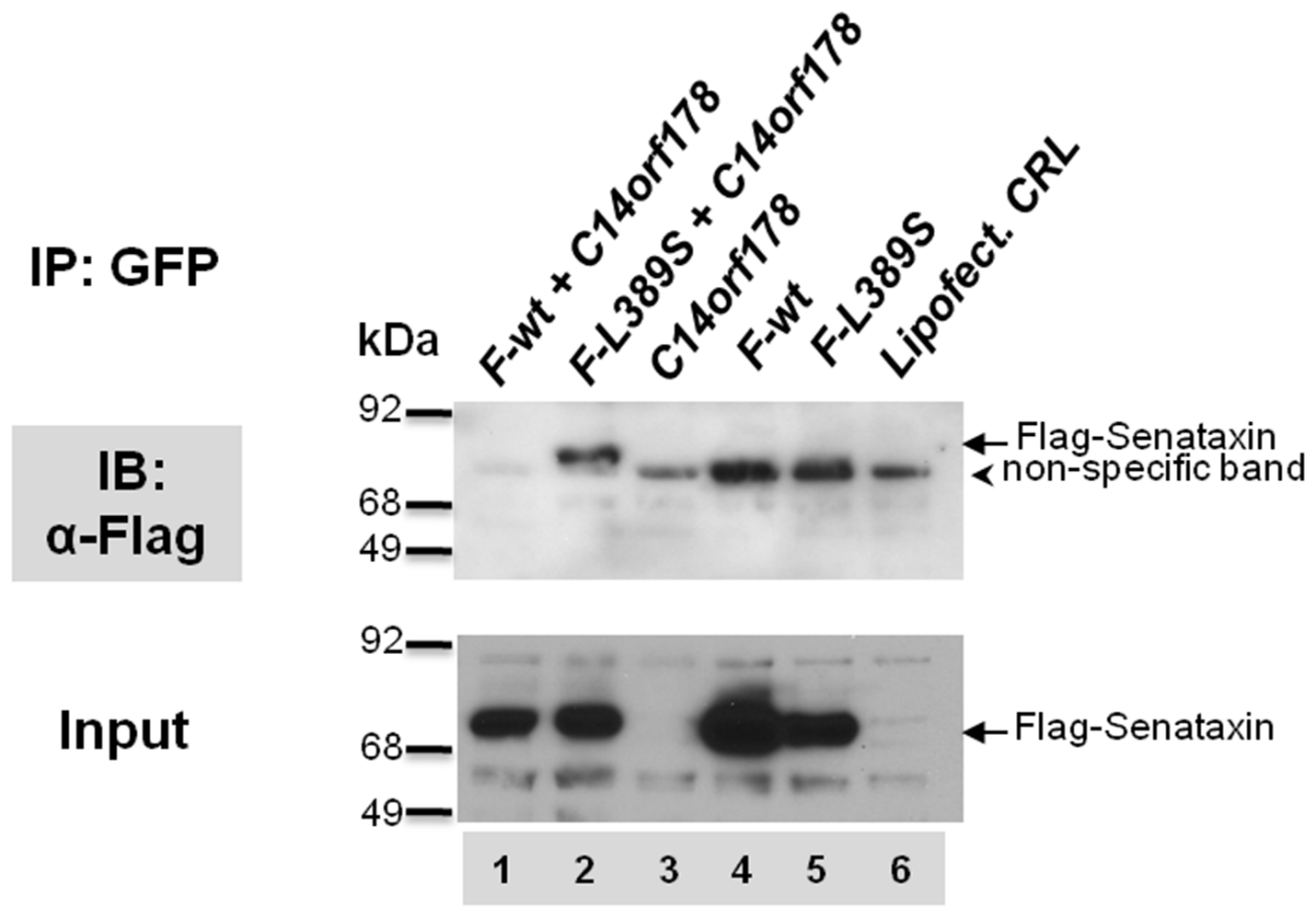
HEK293 cells transfected with expression constructs of Flag-wt-*SETX* (*F-wt*); Flag-L389S-*SETX* (*F-L389S*); and C14orf178-GFP (C14orf178). In the upper panel, proteins isolated upon GFP immunoprecipitation were immunoblotted with anti-Flag antibody, and a band of ∼75 kDa (arrow) representing Flag-Senataxin is detected, but only when Flag-L389S-SETX is co-transfected with C14orf178-GFP. No interaction is detected for Flag-wt-SETX with C14orf178-GFP. In the lower panel, representing ‘Input’, anti-Flag signal at ∼ 75 kDa (arrow) is detected in lanes 1, 2, 4, and 5, as expected for HEK293 cells transfected with recombinant Flag-tagged senataxin protein, prior to anti-GFP IP. In the upper panel, a non-specific band of variable intensity (arrowhead) is detected by anti-Flag antibody in the GFP immunoprecipitates, and is not Flag-senataxin, as it is present in HEK293 cells not expressing Flag-tagged senataxin, and likely corresponds to IgG heavy chain.

To determine if L389S-senataxin would interact with human proteins containing sequence similar to the *BCYRN1-rc* peptide, we obtained a C14orf178 GFP-tagged isoform1 expression construct. After we cloned the amino-terminal senataxin (both -wt and -L389S) into a mammalian expression construct, with the inclusion of a FLAG tag, we co-transfected senataxin-wt or senataxin-L389S with C14orf178-GFP. Western blot analysis confirmed expression of Flag-tagged recombinant senataxin protein. When we immunoprecipitated the C14orf178 expression product with an anti-GFP antibody, we found that Flag-tagged senataxin containing the L389S mutation is pulled down by C14orf178-GFP, but wild-type senataxin is not pulled down by C14orf178-GFP (**Fig. 6**). When we immunostained co-transfected HEK293 cells, we noted that both FLAG-L389S-senataxin and *C14orf178*-GFP localize to the nucleus (data not shown).

## Discussion

We have attempted to shed light on the senataxin protein to understand which of its functions are critical to neuron survival. Despite the obvious link with nucleic acid processing indicated by the presence of a conserved DNA/RNA helicase, the nature of senataxin loss-of-function responsible for AOA2 neurodegeneration remains unknown. Similarly, the molecular basis of senataxin gain-of-function neurotoxicity in ALS4 also remains enigmatic. To remedy this, we pursued an unbiased analysis of the senataxin interactome using a yeast two-hybrid approach, and performed a protein interaction screen with both wild-type senataxin protein and L389S senataxin as the most common ALS4 mutation. Our results revealed several unknown aspects of senataxin biology. First, evidence in support of senataxin dimerization was obtained. Direct analysis of self-association by mammalian two-hybrid (M2H) interaction experiments and by disuccinimidyl suberate cross-linking confirmed senataxin self-association. The L389S mutation did not block dimer formation, but a reduced preference for this state is suggested by the more sensitive M2H analysis.

It is important to emphasize that the ALS4 L389S substitution and all AOA2 missense mutations within senataxin’s amino-terminal protein interaction domain occur at residues that are 100% conserved in all vertebrate species from zebrafish (XP_690945) to man. Indeed, Sen1p protein levels are tightly regulated by a post-transcriptional mechanism requiring the amino-terminal domain [39]. While similar regulation at the protein level has not been shown for mammalian senataxin, high-level protein expression of only truncated senataxin fragments, but not full-length senataxin, can be achieved in mammalian cells [3]. As post-transcriptional regulation of Sen1p involves the ubiquitin proteasome system, our discovery of an interaction between mammalian senataxin and ubiquitination - SUMOylation pathway factors suggests that regulation by this pathway may be conserved between yeast and mammals. We confirmed the functional significance of these putative interactions when we found that NEM treatment of protein extracts (to prevent sumo/ubiquitin cleavage) yielded an ∼7 kDa shift in the molecular mass of senataxin. Although an ∼7 kDa shift is smaller than expected for a single SUMO or ubiquitin addition and could instead represent phosphorylation, the complete elimination of the modification upon NEM treatment makes sumoylation or ubiquitination most likely, as the small shift could represent the migration characteristics of modified senataxin protein. Future study into the nature of senataxin post-translational modification is clearly needed to define the type and number of senataxin post-translational modifications. Bioinformatics analysis indicates that AOA2 or ALS4 point mutations in senataxin do not coincide with predicted sites of ubiquitination or sumoylation; nonetheless, a mono-ubiquitination or sumoylation modification could be important for understanding senataxin normal function, involving processes such as subcellular localization [26].

The DNA/RNA processing proteins (group 3) and homeodomain interacting protein-kinase 2 (group 4) interactors identified by the screen also provide important clues to the normal function of senataxin. Interestingly, the W305C AOA2 loss-of-function mutant, used in the post Y2H screen analysis was also able to bind all 13 wild type senataxin protein interactors. The significance of this is unclear, but one possible explanation is that AOA2 missense *SETX* mutations may yield unstable, reduced protein levels in mammalian cells, a hypothesis that remains untested due to the absence of reliable senataxin Ab’s. Nonetheless, lack of interaction between W305C senataxin and BCYRN1-rc indicates the interaction between L389S senataxin and BCYRN1-rc is specific, and may represent the type gain-of-function interaction expected from a dominant *SETX* mutation. *BCYRN1* is part of the brain cytoplasmic RNA class and shows a number of unique features: (i) it is found only in primates [40]; (ii) it is specifically expressed in neurons and transported to postsynaptic dendrites [46]; (iii) it is derived from a signal recognition particle RNA involved in translation arrest [41]–[44]; (iv) it interacts with poly(A)-binding protein a regulator of translation initiation [47]; and (v) it is found to interact with FMRP, binding with several FMRP mRNA targets via direct base-pairing [48]. Data mining with the BCYRN1-rc translation product revealed strong homology to several proteins, including PPT1 and C14orf178. Further experiments indicated that L389S-senataxin can specifically interact with C14orf178 in mammalian cells, while wild-type senataxin did not. Although further studies are needed to determine if differential senataxin binding to the C14orf178 protein product is relevant to ALS4 disease pathogenesis, evidence of this interaction indicates that the L389S mutation may promote disease pathology by aberrant binding to normally off-target proteins. *BCYRN1* is divided into three domains: (i) a 5′ portion homologous to the Alu Lm (Left Monomer); (ii) a central adenosine-rich region; and (iii) a terminal 43-nt non-repetitive domain [49]. The Alu Lm portion has homology to the Alu repeat element common in the primate genome, and contains the sequence that in reverse complement encodes the 21 amino acid peptide which ALS4 L389S senataxin binds. Alu sequences will inevitably be present in the reverse orientation ∼50% of the time. Given the growing awareness of complex human RNA transcription and multiple regulatory RNA mechanisms [50], [51], it is highly possible that peptide sequences similar to that isolated from our comparative Y2H screen could be generated, even if their translation was unintended. For example, a new toxic mechanism is gaining acceptance as the cause of sporadic and familial ALS based on *C9orf72* hexanucleotide repeat expansions. It has been found that non ATG-mediated translation occurs when the GGGGCC expansion within the normally untranslated intron exceeds a certain limit, thereby generating toxic poly-(Gly-Ala) peptides [52], [53]. Further studies are required to explore this potentially toxic mechanism that we suggest may occur in ALS4 patients based on aberrant L389S senataxin interactions.

This study has uncovered new avenues of research for senataxin biology and ALS4/AOA2 associated mutations. But undoubtedly, many questions remain unanswered. For example, why did a protein such as C14orf178 not emerge during our Y2H screen? One answer is that C14orf178 may not be highly expressed in brain, and based on our analysis of UniGene clusters, C14orf178 was only expressed in rare cancer derived cDNA clones. Yet there are examples that reversed Alu derived sequences find their way into the human transcriptome. The *PPT1* isoform identified by the Wellcome Trust Sanger Institute, 13-JAN-2009 (CAI11026, 2009), has an 87 bp alternate exon 2 which encodes a near perfect match to our ALS4 interacting fragment with the addition of just eight amino acids. Nevertheless, any mechanism put forward to potentially address the ALS4 neuronopathy should provide flexibility to account for the highly variable age of disease onset. If ALS4 senataxin were to retain most normal functions (as in our study it also bound all the same interactors as wild type senataxin), but through point mutation gained the ability to bind ‘abnormally generated’ proteins; this would be an example of the type of model flexibility needed to account for the broad range of disease onset associated with the L389S substitution in ALS4 patients.

## Conclusions

In summary, our study of the senataxin interactome revealed a number of novel aspects of senataxin biology. We report for the first time strong evidence for senataxin self-association and for ubiquitin-SUMO post-translational modification of senataxin, potentially through a set of modifying enzymes and factors from these pathways. For most of the interactions that we detected, both wild type and ALS4 L389S senataxin engaged in these protein interactions. As numerous recent reports have uncovered crucial roles for mammalian senataxin, including the regulation of circadian rhythm and microRNA production from the microprocessor complex, our findings could further advance the understanding of the role of senataxin in these important processes [54]–[56]. Whether mutations in senataxin, such as the L389S alteration, lead to ALS4 motor neuron degeneration by affecting senataxin function in these processes, or promote disease pathogenesis by engaging in an entirely novel and unexpected set of interactions with small peptide products generated from the transcription of primate-specific repetitive elements will be the focus of future studies.
